# The Role of M2 Macrophages in the Progression of Chronic Kidney Disease following Acute Kidney Injury

**DOI:** 10.1371/journal.pone.0143961

**Published:** 2015-12-02

**Authors:** Myung-Gyu Kim, Sun Chul Kim, Yoon Sook Ko, Hee Young Lee, Sang-Kyung Jo, Wonyong Cho

**Affiliations:** Department of Internal Medicine, Korea University Medical College, Seoul, Korea; INSERM, FRANCE

## Abstract

**Introduction:**

Acute kidney injury (AKI) is a major risk factor in the development of chronic kidney disease (CKD). However, the mechanisms linking AKI to CKD remain unclear. We examined the alteration of macrophage phenotypes during an extended recovery period following ischemia/reperfusion injury (IRI) and determine their roles in the development of fibrosis.

**Methods:**

The left renal pedicle of mice was clamped for 40 min. To deplete monocyte/macrophage, liposome clodronate was injected or CD11b-DTR and CD11c-DTR transgenic mice were used.

**Results:**

Throughout the phase of IRI recovery, M2-phenotype macrophages made up the predominant macrophage subset. On day 28, renal fibrosis was clearly shown with increased type IV collagen and TGF-β. The depletion of macrophages induced by the liposome clodronate injection improved renal fibrosis with a reduction of kidney IL-6, type IV collagen, and TGF-β levels. Additionally, the adoptive transfer of the M2c macrophages partially reversed the beneficial effect of macrophage depletion, whereas the adoptive transfer of the M1 macrophages did not. M2 macrophages isolated from the kidneys during the recovery phase expressed 2.5 fold higher levels of TGF-β than the M1 macrophages. The injection of the diphtheria toxin into CD11b or CD11c-DTR transgenic mice resulted in lesser depletion or no change in M2 macrophages and had little impact on renal fibrosis.

**Conclusion:**

Although M2 macrophages are known to be indispensible for short-term recovery, they are thought to be main culprit in the development of renal fibrosis following IRI.

## Introduction

Epidemiologic studies suggest that acute kidney injury (AKI) is the most important precipitating factor in the progression of chronic kidney disease (CKD) [1, 2]. This has been supported by the observations that IRI or nephrotoxic injury in animal models led to tubulointerstitial fibrosis [3, 4]. Although the activation of interstitial myofibroblasts and, recently, the tubular epithelial cell growth arrest at the G2-M phase have been shown to play important roles in linking AKI to the CKD transition [5, 6], the precise underlying pathophysiological mechanisms still remain unclear.

Inflammation plays an important role in the pathogenesis of renal IRI [7, 8]. Neutrophils and macrophages rapidly infiltrate the kidneys in the early phase of IRI and execute their innate immune functions, contributing to the kidney injury by producing reactive oxygen species, proinflammatory mediators, and proteases. In contrast to the neutrophils that are soon cleared, macrophages have been shown to persist during the recovery phase, raising the possibility that these cells contribute to fibrosis. Our previous finding that the depletion of macrophages following the injection of liposome clodronate during the extended recovery period significantly attenuated fibrosis in a rat model of IRI supports this [9]. The heterogeneity of the monocyte/macrophage lineage has long been recognized. As tools to differentiate the subtypes of macrophages have become available, the differential roles of macrophages with distinct phenotypes in various injury and repair models are getting more attention. Lee et al. have recently demonstrated that macrophages shift their phenotype from pro-inflammatory M1 to anti-inflammatory, pro-resolving M2 type macrophages, helping kidney repair following IRI [10]. Our group has also demonstrated the important participation of CD11c^+^ cells in the recovery process by showing that the late administration of liposome clodronate during the recovery phase is associated with persistent tubular damage and inflammation [11]. In an adriamycin nephrosis model, both M2a and M2c macrophages have been shown to reduce renal inflammation and tissue injury and to ultimately improve renal fibrosis [12].

Although there have been advances in the understanding of the role of specific macrophage phenotypes and several studies have shown the *in vivo* therapeutic efficacy of regulatory macrophages, the exact role of macrophages with different phenotypes in the AKI-to-CKD transition remains unclear. Considering the possibility that macrophages can become a therapeutic target or tool in AKI or in the AKI-to-CKD progression, it is necessary to understand the exact role of macrophages with different phenotypes in the AKI-to-CKD model. Therefore, in this study, we examined the alteration of macrophage phenotypes during the extended recovery period following IRI and we determined their roles in the development of renal fibrosis in a mouse model of IRI-CKD.

## Materials and Methods

### Experimental animals and renal IRI

Six- to eight-week-old male C57BL/6 mice (weight, 20~25 g) were purchased from Orient (Seongnam, Korea). The CD11c-DTR B6.FVB-Tg (Itgax-DTR/green fluorescent protein [GFP]; stock number, 004509) and CD11b-DTR B6.FVB-Tg (itgam-DTR/EGFP) stock number, 6000) mice were purchased from the Jackson laboratory (Bar Harbor, ME, USA). All experimental protocols were approved by the animal care committee of the Korea University and followed the NIH publication ‘Principles of Laboratory Animal Care’. To induce IRI, mice were anesthetized with the intraperitoneal (i.p.) injection of 15 mg/kg of ketamine and 2.5 mg/kg of xylazine and subjected to unilateral renal pedicle clamping for 40 min. The animals were kept at a constant body temperature (37°C) using a warm pad. After the clamps were removed, the reperfusion of kidneys was observed for 1 min. A sham operation was performed in a similar manner, except for the clamping of the renal pedicles. To deplete the monocytes/macrophages, liposome clodronate (LC; 10 μl/g) was intravenously administered into C57BL/6 mice, and the diphtheria toxin (DT; CD11c-DTR 4 ng/g, CD11b-DTR first 25 ng/g then 10 ng/g, 10 ng/g, 5 ng/g) was intraperitoneally given to CD11c/CD11b-DTR transgenic mice starting on day 3 after the reperfusion, during 3 weeks at four different time points (day 3, 10, 17 and 24). Mice were sacrificed on days 3, 7, 14, and 28, and the extent of renal fibrosis and macrophage infiltration was examined.

### FACS analysis

Flow cytometric analyses of kidney cells were performed as previously described [11]. Anti-F4/80-FITC, Anti-CD206-APC, anti-CD45-PE and B7-H4 antibodies were purchased from BD biosciences (San Jose, CA, USA) or from eBioscience (San Diego, CA, USA).

### Biochemical tests and measurement of cytokines and chemokines

The serum creatinine levels were measured using a Hitachi 747 automatic analyzer. The cytokines and chemokines in the kidney tissues were quantified using a cytometric bead array (CBA). A mouse inflammation kit (BD Biosciences) was used according to the manufacturer’s instructions, to simultaneously detect mouse interleukin-12p70 (IL-12p70), tumor necrosis factor-α (TNF-α), interferon-γ (IFN-γ), monocyte chemoattractant protein-1 (MCP-1), IL-10, and IL-6, as previously described.

### Histological analyses

Tubular injuries were assessed in periodic acid-Schiff (PAS)-stained kidney sections. For the immunohistochemical staining, we used rat anti-mouse F4/80 (Serotec, Kidlington, UK) and TGF-β1 (Abcam) antibodies. A total of 8–10 high power fields (HPFs) were captured, and the mean number of positive cells was compared between the groups. Renal fibrosis was assessed by Masson trichrome staining, and the area of fibrosis was expressed as the percentage of the blue-stained area in the renal cortex and the outer medulla.

### Western Blot Analysis

Whole cell extracts were loaded and separated by SDS-PAGE under reducing condition, as previously described. The proteins were detected by antibodies against type IV collagen (Abcam) and fibronetic (Santa Cruz Biotechnology, CA, USA), and were visualized using a horseradish peroxidase-linked goat anti-rabbit IgG (Santa Cruz Biotechnology), which was followed by enhanced chemiluminescence (Amersham, Arlington Heights, IL, USA).

### Real time PCR

For the detection of the levels of iNOS and arginase-1 expression in the kidneys, real time RT-PCR was performed using an iCycler IQ Real time PCR Detection System (Bio-Rad, Hercules, CA, USA). The reference gene (RT2 PCR Primer Set, Applied Biosystems) was 18s.

### Bone marrow isolation and macrophage polarization

Bone marrow cells were isolated from the femur and tibia of 8–10 week old C57BL/6 mice. Cells were differentiated into bone marrow-derived macrophages in culture in RPMI-1640 medium (Gibco Invitrogen), 10% heat-inactivated Fetal bovine Serum (FBS), 1% penicillin streptomycin (all Gibco Invitrogen, Breda, The Netherlands) for 8 days. The differentiated macrophages were harvested and re-cultured for 48 h with the normal medium to be M0, with IFN-γ (100 U/ml, Hycult biotech) to be M1, and with IL-10/ TGF-β (each 10 ng/ml, R&D Systems) to be M2c macrophages. To confirm the polarization of the macrophages, they were analyzed using a flow cytometer by staining with F4/80, B7-H4, and CD206 antibodies, respectively, and their TGF-β expression was examined in the culture supernatants (S1 Fig).

### Statistical Analysis

All data are presented as the mean ± standard error, and were analyzed using a T-test or an analysis of variance with post-hoc tests. A p value <0.05 was considered statistically significant. All analyses were performed using version 17.0 of the SPSS statistical software (IBM Corporation, Armonk, NY, USA).

## Results

### Renal fibrosis following unilateral IRI

**Fig 1:**
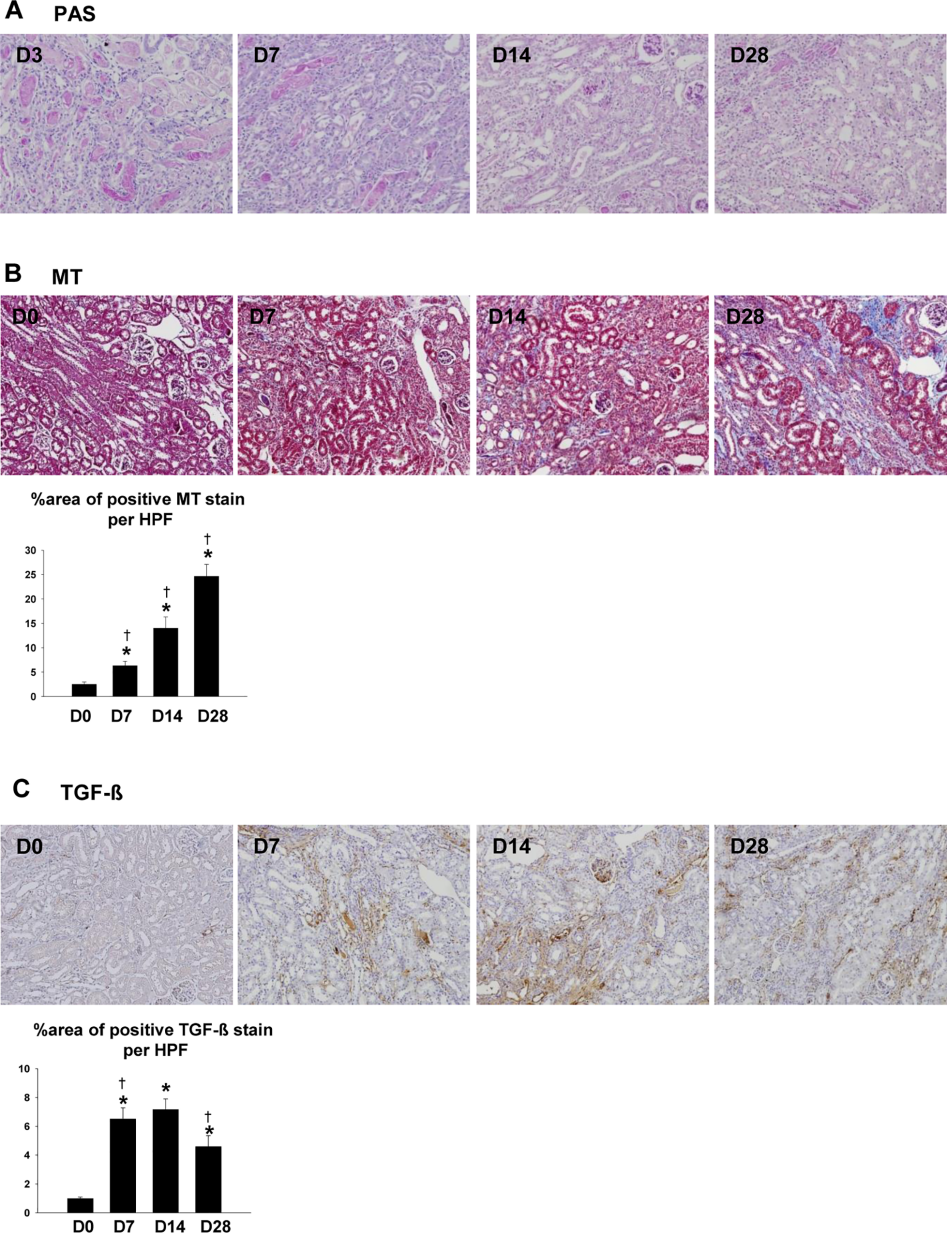
Renal histology following a unilateral ischemia reperfusion injury. (A) As seen by periodic acid-Schiff (PAS) staining, the tubular injury was partially restored from day 3 to day 28 (D3 to D28). (B) As seen by Masson’s trichrome (MT) staining, we observed the development and progression of interstitial fibrosis in the kidneys during the 28 days. (n = 5–6 per group), (C) In immunohistochemical staining, we detected the upregulation of TGF-β. Magnification: ×100, (n = 4–6 per group), *p < 0.05 compared to day 0, ^†^p < 0.05 compared to the previous time point.

To build a model of ischemic AKI-to-CKD progression, we induced a unilateral IRI for 40 min in C57BL/6 mice. Although the repair of post ischemic kidneys was evident from day 7 to day 28 following IRI (Fig 1A), Masson’s trichrome staining showed the development and progression of interstitial fibrosis and tubular atrophy on days 14 and 28 (Fig 1B). In addition, TGF-β, an important cytokine in fibrosis, was also upregulated (Fig 1C).

### Macrophage infiltration during the recovery phase following ischemia reperfusion injury

**Fig 2:**
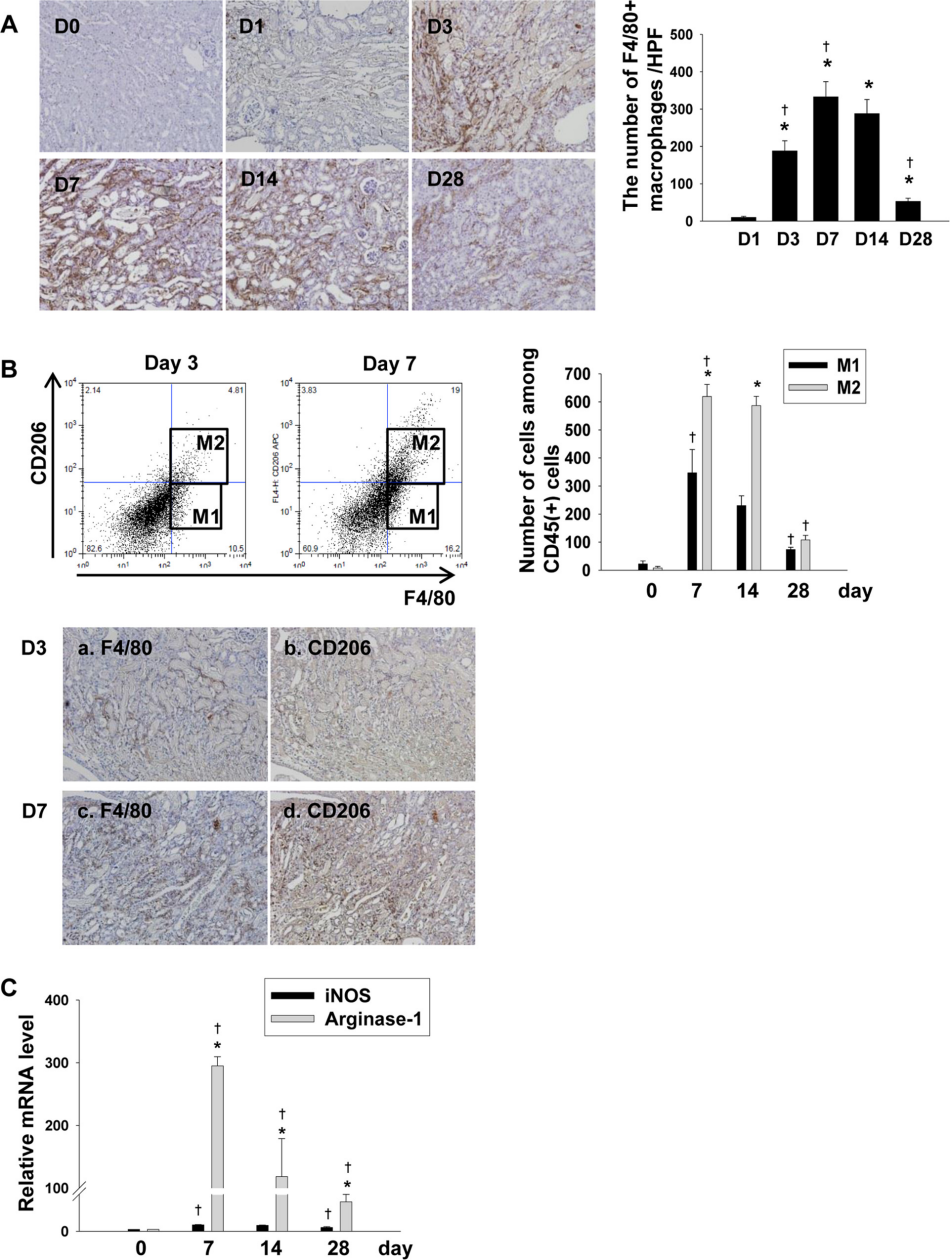
Renal infiltration of macrophages during the recovery phase. (A) In the immunohistochemical staining of F4/80, the amount of F4/80-positive macrophage infiltration increased significantly throughout the recovery phase. Magnification: ×100. (n = 4–5 per group), *p < 0.05 compared to day 1, ^†^p < 0.05 compared to the previous time point, (B) In a flow cytometric analysis to differentiate M1 and M2 macrophages using a CD206 Ab, the macrophage phenotype shifted from being predominantly M1 on day 3 to being predominantly M2 on day 7, 14 and 28 following IRI. (n = 4 per group), *p < 0.05 compared to M1 macrophages, ^†^p < 0.05 compared to the previous time point. As seen by immunohistochemistry, more F4/80-positive cells were shown to be CD206-positive on day 7 (c and d) than on day3 (a and b). Magnification: ×100. (C) An analysis of the mRNA expression level of Arginase-1, an M2 marker, showed a persistently increased expression of more than 100-fold throughout the recovery phase, compared to that of iNOS, an M1 marker. (n = 3–5 per group), *p < 0.05 compared to the mRNA expression of iNOS, ^†^p < 0.05 compared to the previous time point.

The number of infiltrated F4/80^+^ macrophage increased significantly throughout the recovery phase, peaking on day 7, but also showed a persistent increase on day 14, suggesting the possible contribution of macrophages in the development and progression of fibrosis following IRI (Fig 2A). To differentiate M1 and M2 macrophages, we performed a flow cytometric analysis and immunohistochemical staining using a CD206 Ab as a marker of M2 macrophages and found that the macrophage phenotype shifted from being predominantly M1 on day 3 to predominantly M2 on day 7 following IRI (Fig 2B). Likewise, the relative abundance of arginase-1, the M2 marker, far exceeded that of iNOSs, the M1 marker, from day 7 to day 28 (Fig 2C). These results suggest the possibility that M2 macrophages, but not M1 macrophages, play an important role in the development of fibrosis following IRI.

### Preferential M2 Macrophage depletion by liposome clodronate attenuated renal fibrosis

**Fig 3:**
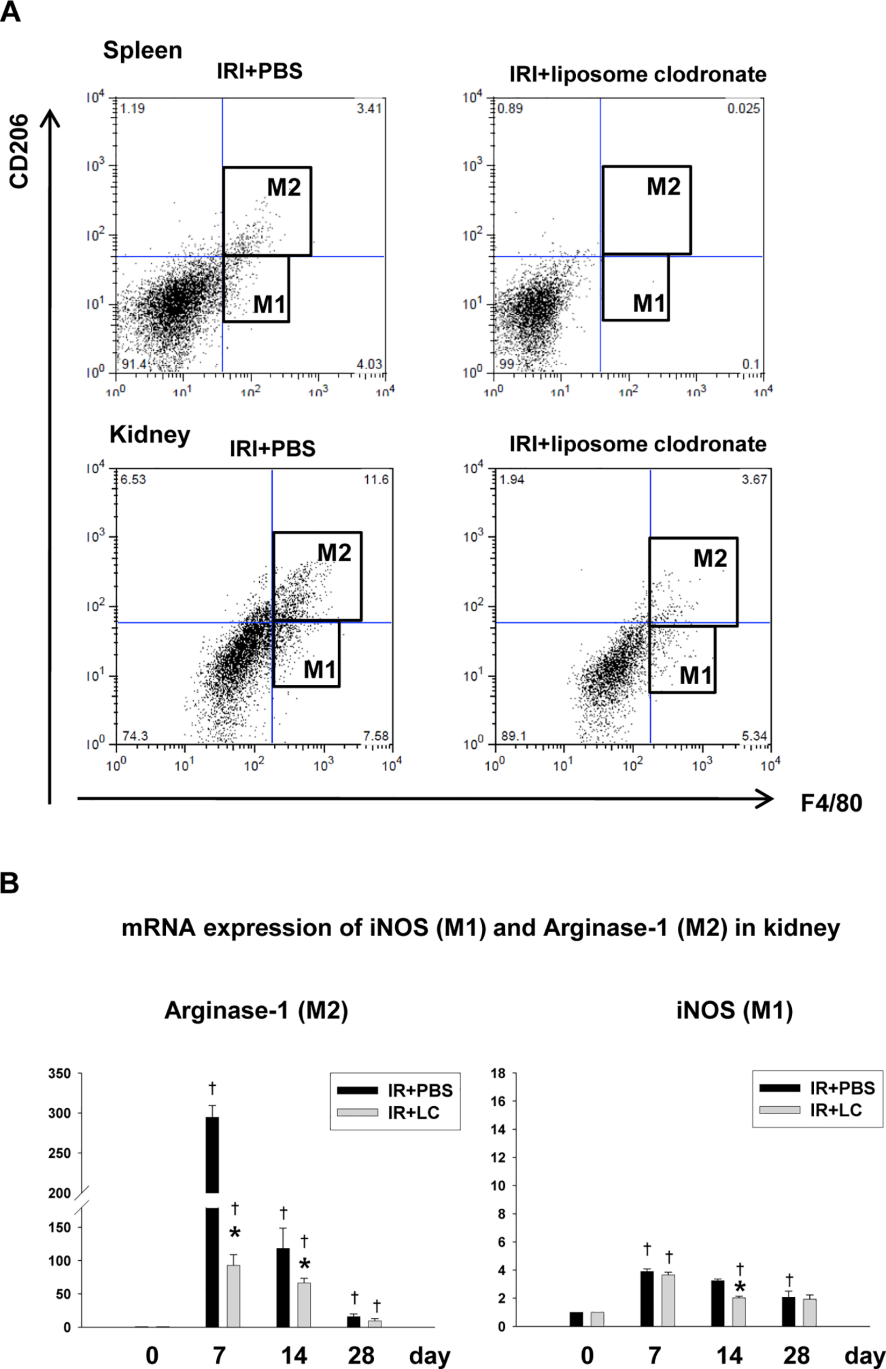
Macrophage depletion by liposome clodronate (LC) administration. (A) In a flow cytometric analysis, both M1 and M2 macrophages were depleted by LC in the spleen and kidneys on day 7 (B) The mRNA expression of the M1/M2 markers showed that the relative reduction of the M2 marker, Arginase-1, was significantly greater than that of the M1 marker, iNOS, in kidneys. (n = 4–6 per group), *p < 0.05 compared to IRI+PBS, ^†^p < 0.05 compared to the previous time point.

**Fig 4:**
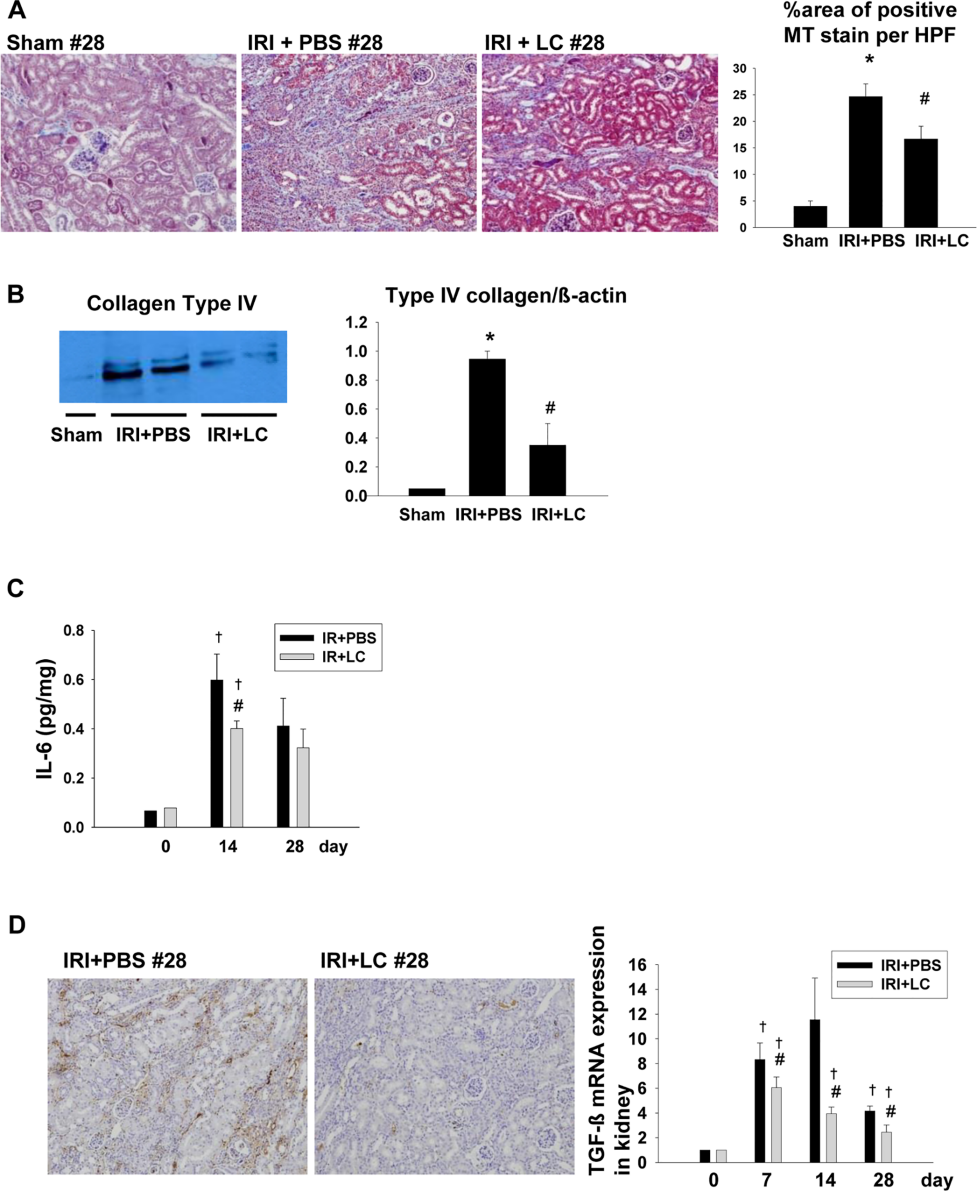
The impact of macrophage depletion on kidneys during the recovery phase. (A) Treatment with LC during the recovery phase improved renal fibrosis (magnification, ×100) and it was associated with a significant reduction in (B) the expression of type IV collagen in kidneys and (C) the level of the pro-inflammatory cytokine IL-6, (n = 3–5 per group), (D) As seen by immunohistochemical staining, the TGF-β expression decreased significantly in the kidneys of clodronate-treated mice on day 28. The TGF-β mRNA expression in kidneys also significantly decreased following liposome clodronate injection during the recovery phase. Magnification: ×100, (n = 4–6 per group), *p<0.05 compared to sham, ^#^p < 0.05 compared to IRI+PBS, ^†^p < 0.05 compared to the previous time point.

To deplete macrophages, we used a liposome clodronate injection. The repeated administration of liposome clodronate starting on day 3 following IRI resulted in the depletion of F4/80^+^ macrophages in the spleen and kidneys. In flow cytometric analyses, the depletion occurred in both M1 and M2 type macrophages (Fig 3A). However, the mRNA expression analysis of the M1/M2 markers showed that the relative reduction of the M2 marker arginase-1 was significantly greater than that of the M1 marker, iNOS, in the kidneys (Fig 3B). We then observed that the depletion of those macrophages during the recovery phase was associated with a significant reduction in pro-inflammatory cytokine IL-6, pro-fibrotic type IV collagen expression and also in the degree of interstitial fibrosis, as assessed at 28 days (Fig 4), suggesting that M2 macrophages play a critical role in the development and progression of fibrosis following IRI. In addition, TGF-β expression decreased significantly in the kidneys of liposome clodronate-treated mice during the recovery phase, in parallel with the macrophage depletion (Fig 4D).

### Adoptive transfer of M2 macrophages, not M1 macrophages, reversed the effect of liposome clodronate treatment on fibrosis

**Fig 5:**
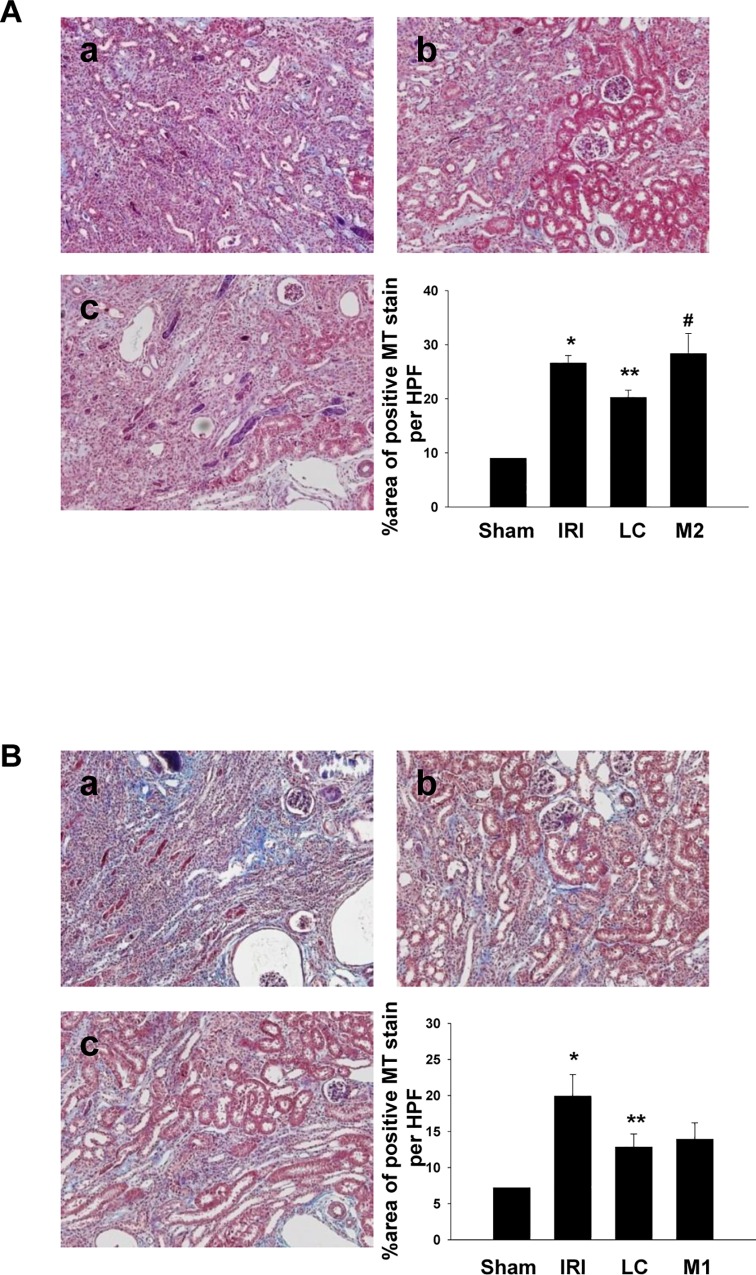
Adoptive transfer of M1 or M2c macrophages following liposome clodronate (LC) treatment. (A) As seen by Masson’s trichrome staining, transferring M2c cells following the LC treatment led to the complete reversion of the improvement of interstitial fibrosis, a. IRI+PBS (IRI), b. liposome clodronate (IRI+LC), c. M2 (IRI+LC+M2c) Magnification: ×100, (n = 4–5 per group), (B) As seen by Masson’s trichrome staining, transferring M1 macrophages into LC-treated mice did not affect the degree of fibrosis, a. IRI+PBS (IRI), b. liposome clodronate (IRI+LC), c. M1 (IRI+LC+M1) Magnification: ×100, (n = 4–5 per group), *p < 0.05 compared to sham, **p < 0.05 compared to IRI, ^#^p < 0.05 compared to LC.

To gain a more insight about the role of M1 and M2 type macrophages in the development of fibrosis, we adoptively transferred the *in vitro* differentiated M1 or M2c macrophages. First, M2 macrophages were differentiated by IL-10/TGF-β and were administered once a week for four weeks following the liposome clodronate injection. The differentiation of bone marrow-derived macrophages into M2c was confirmed by the higher expression of B7-H4, a regulatory surface molecule, and of CD206 by flow cytometry, as well as by the higher TGF-β levels in the culture supernatant compared to that in the M1 or M0 cells (S1 Fig). Transferring these M2c cells following liposome clodronate treatment led to almost complete reversion of the improvement of interstitial fibrosis (Fig 5A). However, transferring M1 macrophages that had differentiated by IFN-γ into liposome clodronate-treated mice did not affect the degree of fibrosis (Fig 5B). Taken together, these results suggest the important role of M2 macrophages, and not M1 macrophages, in the development and progression of fibrosis following IRI.

### M2 macrophages represent a major source of TGF-ß

**Fig 6:**
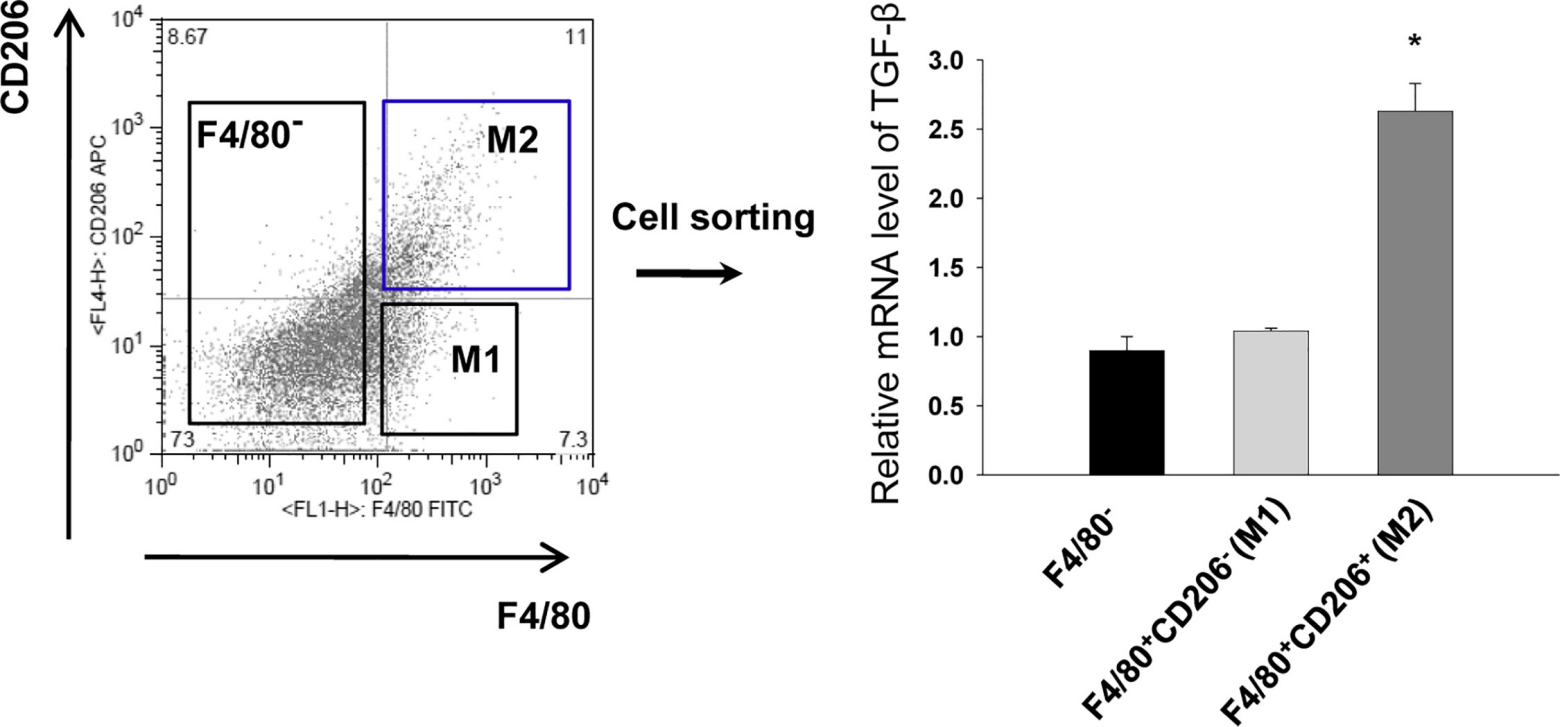
TGF-β mRNA expression of M1 and M2 macrophages. We used a FACs aria analysis to sort M1 and M2 macrophages and compared their TGF-β mRNA expression. The expression of TGF-β was about 2.5 fold higher in M2 macrophages than in M1 macrophages. (n = 4 per group), *p < 0.05 compared to F4/80^+^CD206^-^ M1 macrophages.

To better define whether M2 macrophages represent the main source of TGF-β during renal fibrosis, we used a FACS aria analysis to sort M1 (F4/80^+^CD206^-^) and M2 (F4/80^+^CD206^+^) macrophages from kidneys, and compared their TGF-β mRNA expression using real time RT-PCR. The expression of TGF-β was about 2.5 fold higher in M2 macrophages than in M1 macrophages, confirming that M2 macrophages represent a major source of TGF- β (Fig 6) in CKD transition following IRI.

### Depletion of CD11c^+^ or CD11b^+^ cells using CD11c- or CD11b-diphtheria toxin receptor (DTR) mice had minimal impact on renal fibrosis

**Fig 7:**
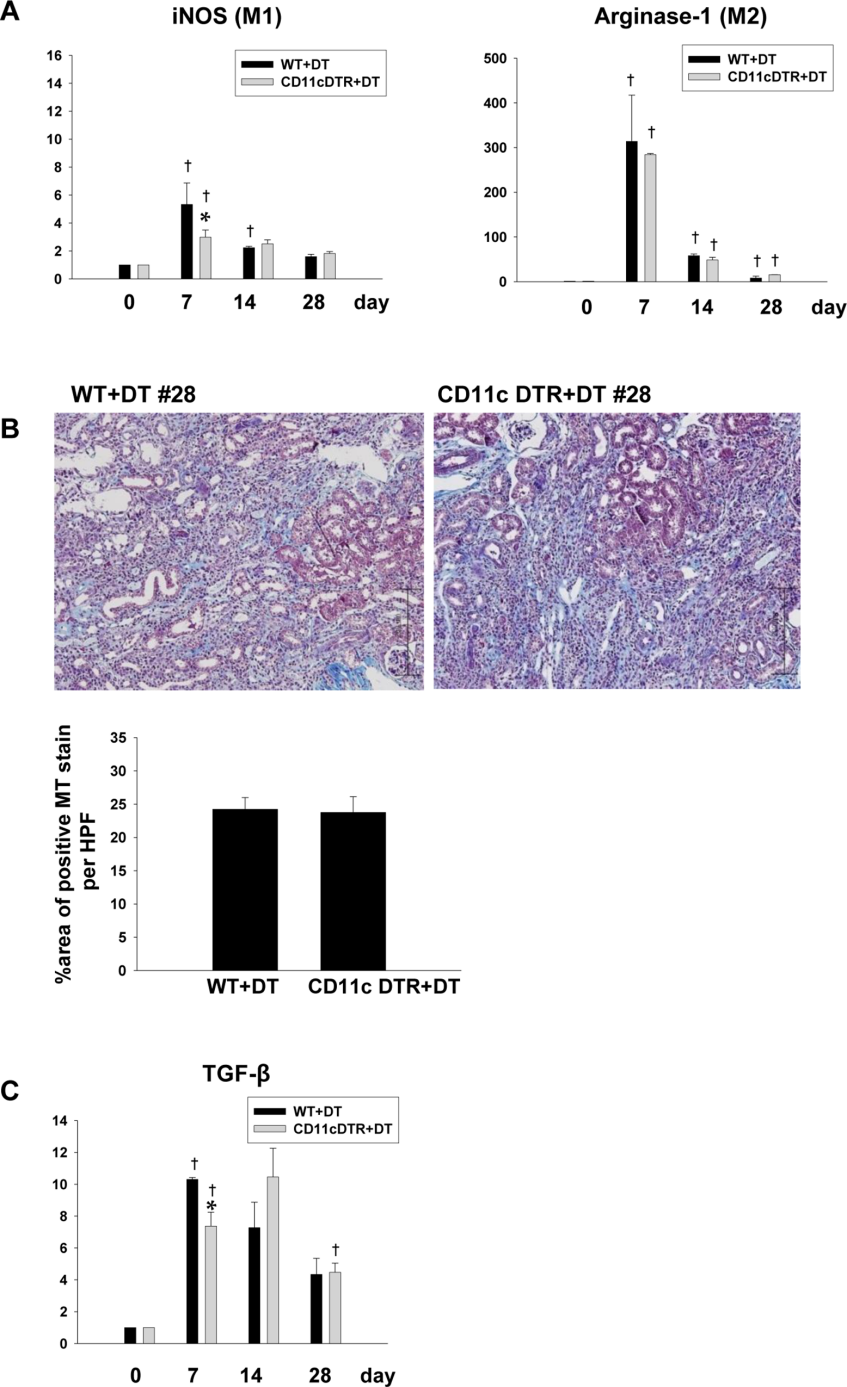
Diphtheria toxin injection in CD11c DTR transgenic mice. (A) An RT-PCR analysis showed no significant difference in the level of mRNA expression of arginase and iNOS in the kidneys. (n = 4–5 per group), (B) Diphtheria toxin injection resulted in no significant change in renal fibrosis on day 28, as seen by Masson’s trichrome staining and (C) in TGF-β mRNA expression throughout the recovery phase. Magnification: ×100. (n = 4–5 per group), *p < 0.05 compared to WT+DT, ^†^p < 0.05 compared to the previous time point.

As macrophages express both CD11b and CD11c, we also tested the effect of the depletion of CD11b or CD11c on the development of fibrosis following IRI. As expected, diphtheria toxin (DT) injection into CD11c DTR mice resulted in the depletion of CD11c^+^ cells in the spleen and kidneys (data not shown). However, unlike the response to the administration of liposome clodronate, the depletion of CD11c^+^ cells did not alter the expression level of arginase (Fig 7A), and this was associated with a minimal effect on TGF-β expression as well as on the degree of fibrosis (Fig 7B and 7C).

**Fig 8:**
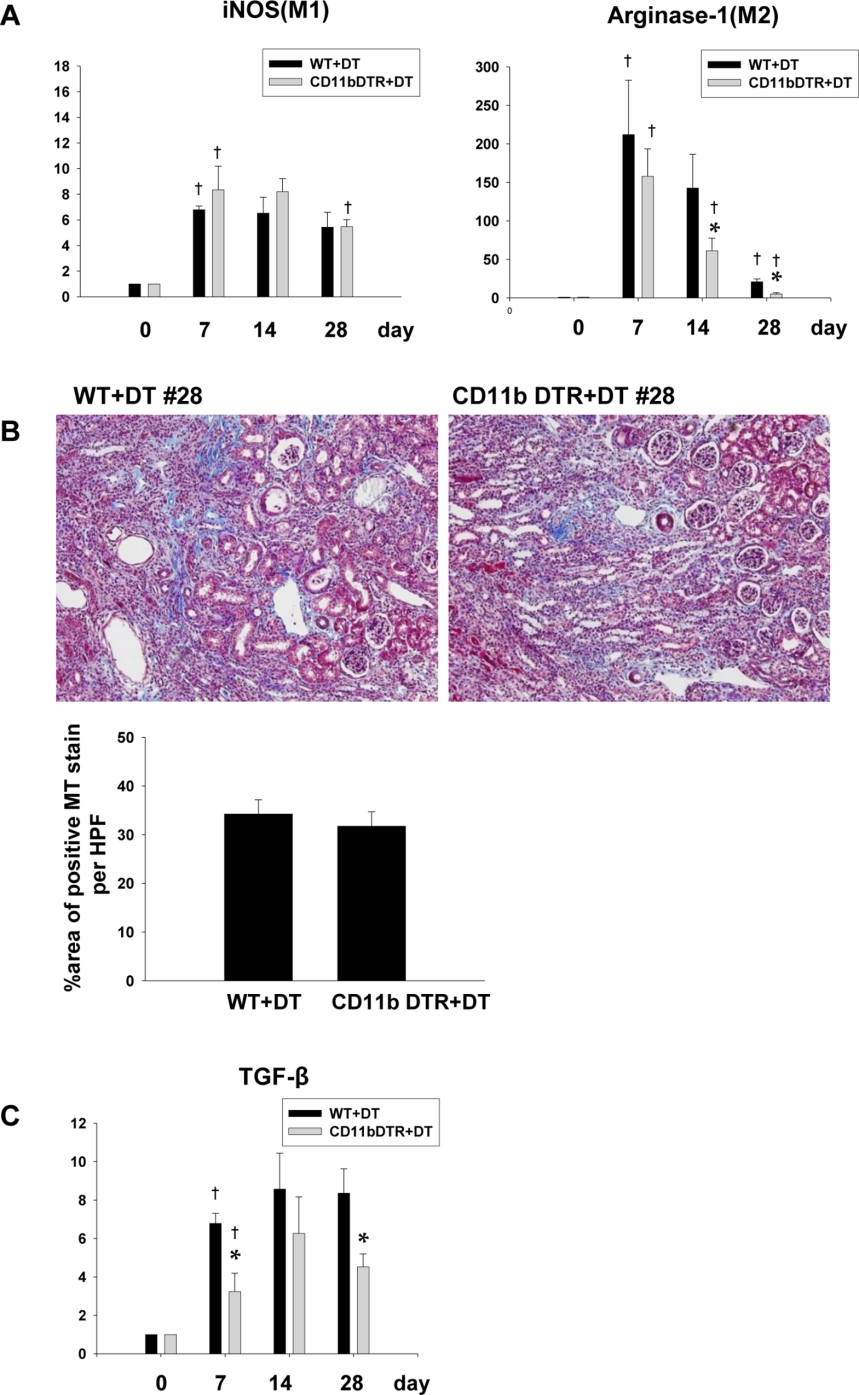
Diphtheria toxin injection into CD11b DTR transgenic mice. (A) An RT-PCR analysis showed the relative reduction in the mRNA expression level of arginase-1. However, on day 7, it was less significant than the reduction observed in kidney of liposome clodronate-treated mice. (n = 4–5 per group), (B) Diphtheria toxin injection resulted in no significant change in renal fibrosis on day 28, as seen by Masson’s trichrome staining, and (C) in TGF-β mRNA expression throughout the recovery phase. Magnification: ×100. (n = 4–5 per group), *p < 0.05 compared to WT+DT, ^†^p < 0.05 compared to the previous time point.

In CD11b DTR mice, the DT injection resulted in the depletion of CD11b^+^ cells in the kidneys. Interestingly, the DT injection in the CD11-b DTR system resulted in the preferential depletion of M2 type macrophages, such as liposome clodronate. However, percent decrease on day 7 when M2 infiltration was most predominant in the kidney, was significantly lower compared to that of liposome clodronate and this led to failure of reversal of renal fibrosis (Fig 8).

## Discussion

In this study, we demonstrated that pro-fibrotic M2 macrophages become predominant in kidneys during the recovery phase and that they play a critical role in the development and progression of fibrosis after IRI.

The prevalence of CKD in the Unites States exceeds 10% and is expected to grow at the fastest rate worldwide [13]. As with CKD, AKI is also being increasingly recognized in critically ill patients, being an independent predictor of mortality and substantial proportion of patients failed to achieve complete renal recovery [1, 2, 13]. Considering that AKI is the most important precipitating factor of CKD progression, strategies to facilitate AKI recovery could help prevent progressive CKD and its poor outcomes. Moreover, if identifying the patients who are at a high risk of progressive CKD following AKI is possible, measures to slow the progression of CKD can be implemented at earlier time points according to the patient's expected prognosis. However, little is known about the precise pathophysiological mechanisms underlying the AKI-to-CKD transition, and there is currently no tool available to promote AKI recovery or to prevent CKD progression following AKI. Understanding the mechanisms underlying the transition from AKI to CKD can start with inflammation, because AKI is an inflammatory disease and also many chronic diseases, such as arthritis, diabetes, and connective tissue diseases, are characterized by chronic persistent inflammation.

Neutrophils and macrophages are well known to infiltrate the kidneys and play an important role in early injury by producing multiple tissue-destroying mediators [7, 8]. In contrast to the neutrophils that are cleared from the kidneys, a recent study by Lee et al. showed that the kidney-infiltrating macrophages persisted throughout the recovery phase and shifted their phenotype from iNOS-producing pro-inflammatory M1 type macrophages to arginase-producing anti-inflammatory, pro-resolving M2 type macrophages [10]. The late depletion of macrophages during the recovery phase significantly impairs recovery, supporting a function of M2 macrophages that is opposite to that of M1 macrophages, thus playing an important role in the repair process. Although M2 macrophages, which produce not only arginase but also IL-10 and TGF-β are thought to be necessary for kidney repair, their fate inside the kidneys or their long-term effect on fibrosis remains uncertain. Considering that *in vitro*-differentiated M2a or M2c macrophages express TGF-β [12], an important fibrosing cytokine, it is possible that kidney M2 macrophages critically contribute to the development of fibrosis following IRI. In animal models of renal fibrosis, such as Alport syndrome and unilateral ureteral obstruction (UUO), M2 macrophages are the predominant macrophage subtype [14, 15]. The pro-fibrotic role of M2 macrophages has also been demonstrated in several other fibrosis models. In a murine model of pulmonary fibrosis, an alternative activation of M2 macrophages was shown as an important signaling pathway in the pathogenesis of lung fibrosis [16, 17]. The observation that enhanced fibrosis in the surrounding tissue of Myd88 KO mice with a staphylococcal catheter infection was accompanied by the recruitment of M2 macrophages could also support the important participation of M2 macrophages in fibrosis [18]. However, there are some conflicting results about the role of M2 macrophages. During the IRI recovery phase, in which inflammation is only transient and short, M2 macrophages act mainly as scavengers of apoptotic cells and debris, and, if recurrent or persistent injury does not interfere, they may promote the healing process without fibrosis. Some researchers have also concluded that anti-inflammatory macrophages play an important role in the repair of renal fibrosis [10, 12, 19, 20].

In this study, we used three different methods to deplete macrophages, to clearly define their roles in fibrosis progression, because the currently available depletion strategies lack specificity, and there is no way to deplete a single subtype of macrophages. We first observed that macrophage infiltration persisted until four weeks after IRI, and that the M2 macrophages were becoming predominant. We observed a greater abundance of CD206^+^ macrophages by flow cytometry, as well as a huge increase in arginase, but not in iNOS, which support the M2 predominance during the recovery phase. First, we administered liposome clodronate during the time and this resulted in the depletion of both M1 and M2 type macrophages. There were some discrepancies between data of FACs analysis and real time PCR. This might result from the difference in expression levels of the protein and RNA. Moreover, two different detecting markers of the analysis might have caused the discrepancy of results. Although precise mechanism underlying this results are unclear, the predominantly infiltrated M2 macrophages showed a greater percent decrease relative to M1 macrophages, and this was associated with profound renoprotection with regard to fibrosis progression. Alternatively, we used the two types of the macrophage depletion by using mice transgenic for the DTR under the control of the CD11b or CD11c promotor, because the expression of CD11c and CD11b on kidney mononuclear cells is heterogeneous, and there is phenotypic overlap between these cells. Although the injection of DT into CD11b DTR mice also resulted in the depletion of both M1 and M2, the depletion efficiency was far less than that achieved by the injection of liposome clodronate, and this did not affect progressive fibrosis. In addition, the depletion of CD11c^+^ cells using CD11c-DTR mice affected neither the population of M2 macrophages nor the degree of fibrosis. Taken together, these results might suggest the important role of M2 macrophages in the progression of CKD following AKI. However, we can consider a few things as the causes of the difference in the depletion of macrophages between each method. First, the antigen expression of CD11b or CD11c shows heterogeneity on different type of myeloid cells, and these phenotypes of macrophages might be dynamically changed during the recovery phase. Therefore, DT-induced depletion of each type of macrophages can be different depending on the time after IRI. However, in this study, the phenotypical changes with time were not investigated further. Additionally, in CD11b mice, we injected high dose of DT on day3, and then gradually reduced the dose to prevent toxicity. This might result in insufficient depletion of macrophages.

To get better understanding of the pathogenetic role of M2 macrophages in the development and progression of fibrosis following IRI, we adoptively transferred *in vitro*-differentiated M1 or M2 macrophages in clodronate-treated mice. M2c macrophages were used because they are known to be much more similar to the *in vivo* anti-inflammatory macrophages that participate in the repair process than to M2a or M2b macrophages [12]. Whereas the administration of M2c macrophages partially reversed the clodronate-induced beneficial effect on fibrosis, transferring M1 macrophages did not affect fibrosis. This result strongly suggests that it is M2 macrophages that play a critical role in the generation of fibrosis.

TGF-β plays a key role in fibrosis by activating mesangial cells, interstitial myofibroblasts and through the epithelial-mesenchymal transition [21]. In our study, the clodronate-induced depletion of macrophages was associated with a significantly decreased expression of TGF-β in the kidneys. In addition, our observation that TGF-β secretion from M2c macrophages was significantly higher than that from M1 macrophages suggests the critical role of M2 macrophage-producing TGF-β in fibrosis. Therefore, although known to be important in facilitating recovery, the massive or persistent infiltration of M2 macrophages during the resolving process following IRI can be harmful in terms of CKD progression, because the excessive or prolonged production of TGF-β from M2 macrophages might induce the deposition of collagen and other ECM components. Therefore, the unconditional transfusion or induction of M2 macrophages may initiate fibrotic changes in AKI. Indeed, those macrophages could be regarded as a “double-edged sword” in the CKD progression following an AKI. Therefore, maintaining the balance in the activity of M1/M2 macrophages during the repair process should be more carefully conducted to prevent CKD progression. The previous observation that M2 macrophages improved tissue fibrosis in adriamycin-induced nephrosis after having just been transferred by a single injection during an early phase of injury suggests the importance of the appropriate timing of macrophage administration [12]. Although imperative for recovery or repair, a prolonged and skewed balance towards M2 macrophages during an extended period following IRI is thought to exacerbate kidney fibrosis.

## Conclusions

In conclusion, our study showed that M2 macrophages play an important role in fibrosis progression during the AKI-to-CKD transition and that they are, at least in part, dependent on TGF-β. Therefore, when macrophages are considered as a therapeutic target or tool in the AKI-to-CKD progression, a better understanding of the optimal balancing of M1/M2 macrophages to promote recovery and reduce fibrosis is required for the development of clinically applicable strategies for the prevention of CKD progression. In addition, in real clinical practice, various kinds of acute insults can cause the progression to CKD such as nephrotoxic agent, infection and autoimmune diseases as well as ischemic AKI. Therefore, the role of M1 or M2 macrophages in other types of AKI should also be tested.

## Supporting Information

S1 Fig A) Bone marrow-derived macrophages were cultured for 48 h with the normal medium to be M0, with IFN-γ (100 U/ml) to be M1, and with IL-10/ TGF-β (each 10 ng/ml,) to be M2c macrophages and their polarization was examined using a flow cytometer by staining with F4/80 and B7-H4 antibodies, respectively. B) Their TGF-β expression was examined in the culture supernatants. *p < 0.05 compared to M0. (PDF)
