# Supplementary figures and images for: The Role of M2 Macrophages in the Progression of Chronic Kidney Disease following Acute Kidney Injury
Source: PLoS One. 2015 Dec 2;10(12):e0143961. doi: 10.1371/journal.pone.0143961 (PMC4667939; doi:10.1371/journal.pone.0143961)

S1 figure

A

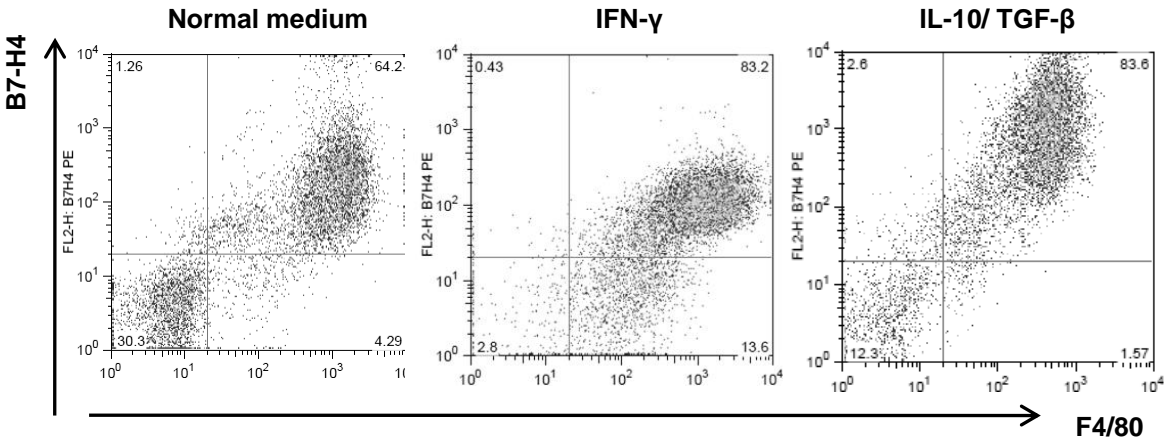

B

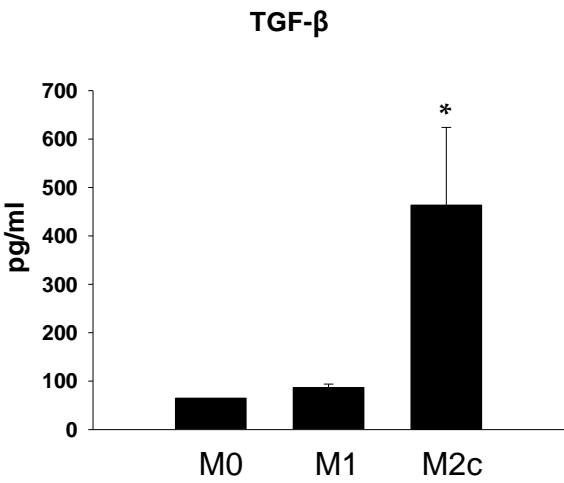

Supplement: S1 Fig — (PDF) [file pone.0143961.s001.pdf]
